# Adaptive fuzzy sliding mode control of an actuator powered by two opposing pneumatic artificial muscles

**DOI:** 10.1038/s41598-023-34491-3

**Published:** 2023-05-22

**Authors:** Minh-Duc Duong, Quang-Thuyet Pham, Tuan-Chien Vu, Ngoc-Tam BUI, Quy-Thinh Dao

**Affiliations:** 1 Hanoi University of Science and Technology, Hanoi, 11615 Vietnam; 2 Shibaura Institute of Technology, Saitama, 337-8570 Japan

**Keywords:** Engineering, Biomedical engineering, Electrical and electronic engineering, Mechanical engineering

## Abstract

Pneumatic artificial muscle (PAM) is a potential actuator in human–robot interaction systems, especially rehabilitation systems. However, PAM is a nonlinear actuator with uncertainty and a considerable delay in characteristics, making control challenging. This study presents a discrete-time sliding mode control approach combined with the adaptive fuzzy algorithm (AFSMC) to deal with the unknown disturbance of the PAM-based actuator. The developed fuzzy logic system has parameter vectors of the component rules that are automatically updated by an adaptive law. Consequently, the developed fuzzy logic system can reasonably approximate the system disturbance. When operating the PAM-based system in multi-scenario studies, experimental results confirm the efficiency of the proposed strategy.

## Introduction

In recent years, the PAM has been one of the most promising actuators for applications requiring the simulation of human-like movements. The PAM consists of a long tube made of rubber and covered with braided yarn. PAM stiffens and contracts in radial and longitudinal directions when supplying compressed air. Conversely, it will soften and lengthen when we release the air. That contraction is similar to the principle of operation of the muscle bundles of living things. PAMs are usually utilized in industrial applications due to their advantages of quick reaction, extremely lightweight, high power-to-weight and power-to-volume ratios, inherent safety, cleanness, ease of maintenance, pliability, and low-cost^1–5^. Some prominent applications include manipulators^4,6–8^ to enhance the safety of humans who interact with robots, rehabilitation systems^9–14^, and medical devices^15,16^ to assist patients in restoring motor function. However, PAM is a nonlinear system with a huge latency, and regulating it with good performance always attracts great attention from researchers.

Furthermore, determining a nonlinear mathematical model of PAM is extremely challenging, resulting in a bias in the estimation of the PAM-based system’s parameters. As a result, PAM-based systems have a lot of unknown disturbances. Many control methods have been proposed to solve the problems of the pneumatic muscle actuator. Many early studies chose the Proportional-Integral-Derivative (PID) controller and its modified versions. A nonlinear PID-based controller^17–21^ for enhancing correction of non-linear hysteresis phenomenon and increased robustness. A fuzzy PID controller^22–25^ is proposed to improve trajectory tracking performance. Most of the mentioned controllers have decent performance. They are inadequate to deal with PAM’s hysteresis and nonlinearity.

To overcome the drawbacks of the PID controller and its improved variants, nonlinear control approaches such as sliding mode control (SMC), dynamic surface control, adaptive control, interactive learning control, and intelligent control have been presented in the literature. More specifically, conventional sliding mode control is applied in Refs.^26,27^ for trajectory tracking of a PAM system. Different types of discrete-time sliding mode control are used for robust position control of a PAM system^28,29^. In addition, dynamic surface control that uses first-order filter to improve the system response is also applied to tracking control of PAM systems^30^. Moreover, In Ref.^31^, the authors recommend adaptive control to estimate unknown system parameters online, which achieves satisfactory control performance.

Interactive learning control and intelligent control that can learn the nonlinearity and estimate unknown parameters are also prominent approaches to controlling the PAM system. The authors of Ref.^32^ proposed a robust iterative learning control algorithm to address a PAM system’s uncertainties and state constraints. Fuzzy control in combination with fractional PID control^25^, with sliding mode control^33^, and with model predict control^34^ are proposed for control of the PAM system. In these articles, fuzzy logic plays a role in adjusting the control parameters. Reference^35^ proposed an adaptive fuzzy sliding mode control approach to regulate a PAM system without a pre-defined model, in which the unknown parameters are estimated using fuzzy functions. Similarly, Ref.^36^ employed the same idea, but instead of fuzzy logic, a neural network was utilized to estimate the unknown functions. Moreover, reinforcement learning is also considered to optimize the control performance of the PAM system^37^. Most of the aforementioned approaches can bring robustness to the system. Some of them try to improve the system performance by estimating the unknown parts and disturbances with very complicated estimation algorithms. These algorithms are theoretically effective, but their implementation is very difficult with much computation. Thus, the requirement for an effective control algorithm is still an open problem.

Based on the favorable research findings of fuzzy and adaptive-based controllers, we tackle the control of a nonlinear PAM system with unknown disturbance by treating it as a linear system with unknown disturbance. We propose an adaptive fuzzy algorithm combined with a sliding mode control law to estimate and compensate for the disturbance while addressing approximation errors and model uncertainties. To enable practical implementation, we design the algorithm in the discrete domain, making it feasible for programming on a digitally embedded device. Our paper makes several contributions to the field of control engineering for nonlinear systems, particularly in the context of PAM systems with unknown disturbances as follow

- Proposes an adaptive fuzzy sliding mode control algorithm to control a nonlinear PAM system with unknown disturbance by considering it as a linear system with unknown disturbance.
- The proposed approach has the advantage of using fuzzy logic to estimate unknown parameters, making it more effective in handling complex and nonlinear systems.
- Designs the AFSMC algorithm in the discrete domain for practical implementation on a digitally embedded device.

## System description

**Figure 1:**
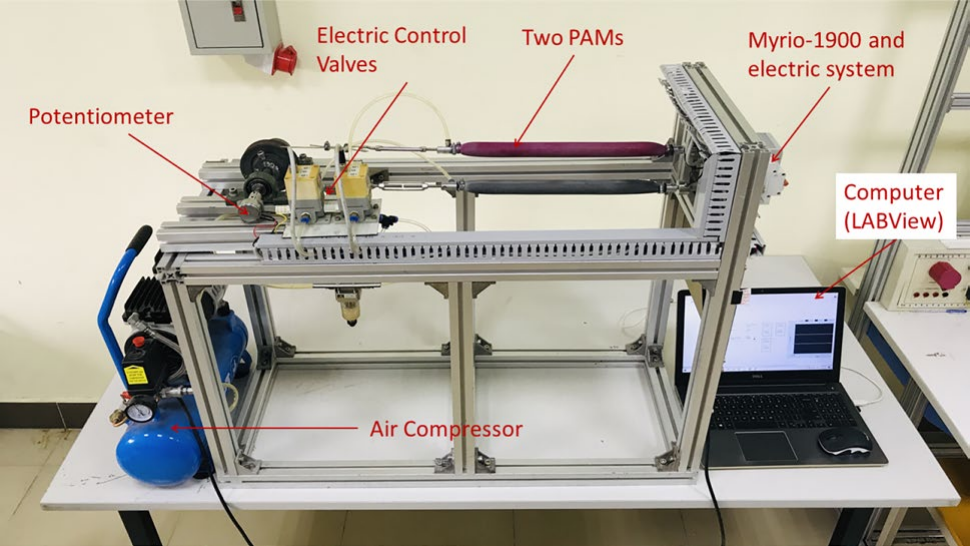
The experiment platform of two opposing PAMs actuator^38^.

The system structure is shown in Fig. 1. The system includes an air compressor that supplies air to two artificial muscle bundles (with $23 \times 10^{-3}$ (m) of diameter, $40 \times 10^{-2}$ (m) of nominal length). When being inflated and deflated to the artificial muscle system through two proportional valves (SMC, ITV-2030-212S-X26), one muscle bundle contracts, and the other relaxes, causing the pulley to rotate around its center. The rotational angle produced is measured by the potentiometer (WDD35D8T). The myRIO-1900 embedded controller from National Instrument was utilized in this experiment to compute the feedback angle from the potentiometer and generate the control signal to the proportional valves, while the LabVIEW software was used for monitoring the entire process.

**Figure 2:**
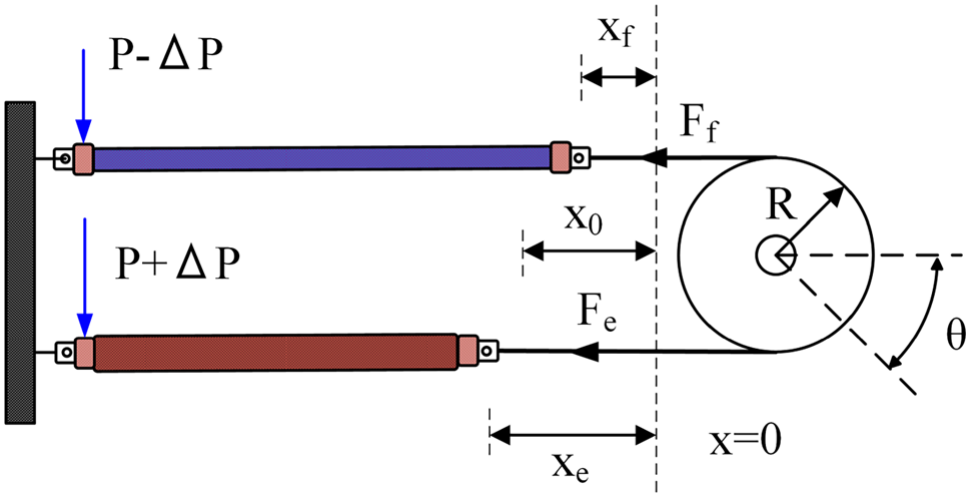
The structure schematic of two opposing pneumatic artificial muscles.

Figure 2 shows a schematic diagram of the working principle of a pneumatic artificial muscle, which describes each relationship between the air pressure, the motion of the artificial muscles, and the deflection angle of the pulley. Initially, the pressure in the muscle bundles is set at $P_{0} = 0.2$ MPa. Equation (1) describes the internal pressure of two PAMs in operation.

1 $$\begin{aligned} {\left\{ \begin{array}{ll} P_1 = P_0 + \Delta P \\ P_2 = P_0 - \Delta P \end{array}\right. } \end{aligned}$$

where $P_1$ and $P_2$ are the pressures of the two PAMs, $P_0$ is the initial pressure, and $\Delta P$ is the pressure difference between the two PAMs. The dynamical model of a single Pneumatic Artificial Muscle (PAM) can be expressed using Reynolds’s model^39^ as:

2 $$\begin{aligned} {M\ddot{x} + B(P)\dot{x} + K(P)x = F(P) - Mg} \end{aligned}$$

with

3 $$\begin{aligned} {\left\{ \begin{array}{ll} K(P) = {K_0} + {K_1}P\\ B(P) = {B_{0j}} + {B_{1j}}P\\ F(P) = {F_0} + {F_1}P \end{array}\right. } \end{aligned}$$

where *x* is the contraction in the length of PAM. The model components representing the spring, damping, and contractile elements are represented by *K*, *B*, and *F*, respectively. ${K_i}$ and ${F_i}$ (i = 0,1) are constants. ${B_{i,j}}$ are linear functions. The value of *j* represents whether the PAM is contracting ($j = 1$) or deflating ($j = 2$). In a configuration where two PAMs act antagonistically, they generate a torque *T* on the pulley, which has an inertia moment *J*. The expression for the torque *T* is as follows:

4 $$\begin{aligned} \begin{aligned} T&=J\ddot{\theta }(t)=(F^{PAM}_{e} - F^{PAM}_{f})r \end{aligned} \end{aligned}$$

where *r* represents the pulley’s radius. The forces $F^{PAM}{e}$ and $F^{PAM}{f}$ created by each PAM can be expressed as:

5 $$\begin{aligned} \begin{aligned} F^{PAM}_{e}= F_{e} - B_{e}{\dot{x}}_{e} - K_{e}x_{e}\\ F^{PAM}_{f}= F_{f} - B_{f}{\dot{x}}_{f} - K_{f}x_{f} \end{aligned} \end{aligned}$$

The contractions of the PAMs $x_e$ and $x_f$ in Eq. (5) can be obtained by using the initial contraction ($x_0$) and the pulley’s angle ($\theta$), as shown below:

6 $$\begin{aligned} x_{e,f}=x_0\pm r\theta \end{aligned}$$

Assuming that the two PAMs have similar mechanical parameters, we can use Eqs. (3), (4), (5), and (6) to derive the following expression:

7 $$\begin{aligned} \begin{aligned} T= 2(F_1-K_1 x_0)r\Delta P(t)- \left[ B_{0e} +B_{0f} + (B_{1e}+B_{1f}){P_0} \right] r^2{\dot{\theta }}(t)- 2 (K_0+K_1P_0)r^2\theta (t) \end{aligned} \end{aligned}$$

or

8 $$\begin{aligned} c_1 \Delta P(t) = \ddot{\theta }(t) + c_2 {\dot{\theta }}(t) + c_3\theta (t) \end{aligned}$$

in which $c_1 = \displaystyle {\frac{2(F_1-K_1 x_0)r}{J}}$, $c_2 = \displaystyle {\frac{\left[ B_{0e} +B_{0f} + (B_{1e}+B_{1f}){P_0} \right] r^2}{J}}$, and $c_3 = \displaystyle {\frac{2 (K_0+K_1P)r^2}{J}}$.

To facilitate the design of the controller on a real-time processor, we consider the following discrete-time formulation of the model (Eq. 8).

9 $$\begin{aligned} y_{k}=-\sum _{i=1}^{n}a_{i}y_{k-i}+\sum _{j=1}^{m}b_{j}u_{k-j}+p_{k}, \end{aligned}$$

The control signal $u_k$ represents the different pressure $\Delta P$ applied to the PAM system, while $y_k$ represents the pulley’s angle deflection $\theta$. The disturbance and unknown uncertainties in the system are denoted by $p_k$, and the model parameters are represented by $a_i$ and $b_j$, $m=n=2$. The identified model parameter values are shown in Table 1.

**Table 1:** System parameters.

Parameters	Values
$a_1$	$-1.9345 \pm 9.2\times 10^{-3}$
$a_2$	$0.9765 \pm 12.8\times 10^{-3}$
$b_1$	$0.0126 \pm 1.3 \times 10^{-3}$
$b_2$	$0.0124 \pm 4.9 \times 10^{-3}$

## Controller design

**Figure 3:**
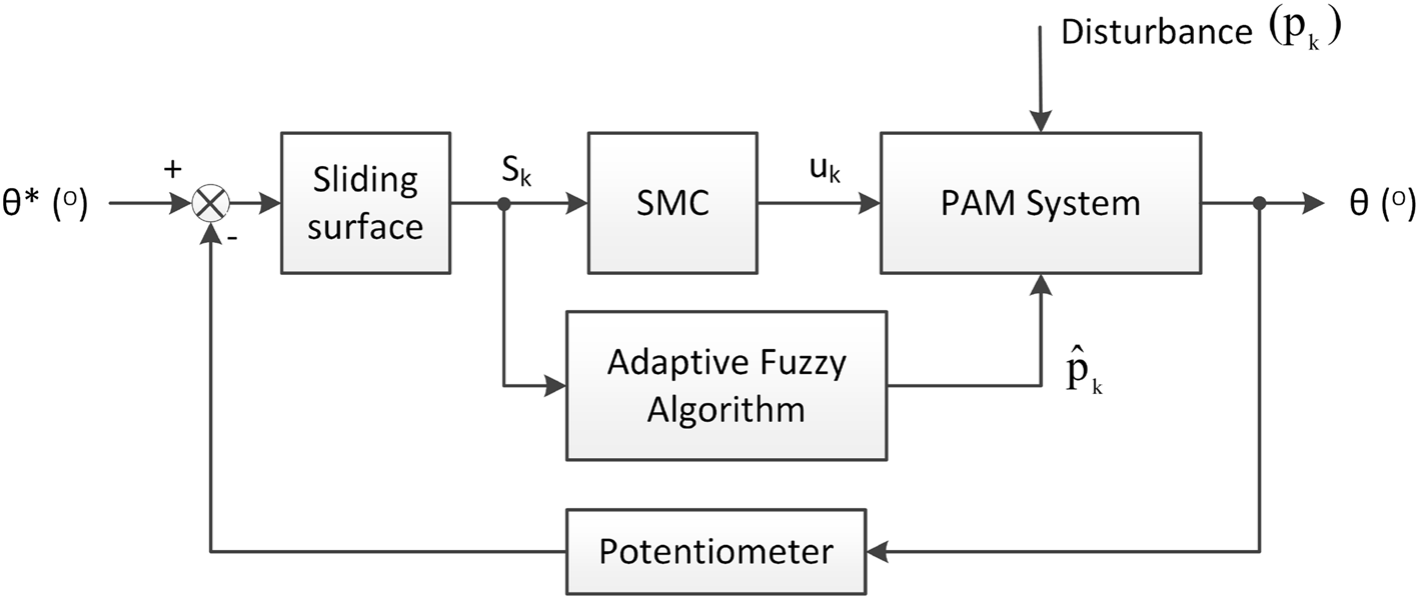
Block diagram of the proposed adaptive fuzzy sliding mode controller.

This section outlines the construction of the proposed AFSMC for the PAM system, which involves several steps. Initially, a sliding mode controller is developed with a control signal containing a variable ${\hat{p}}_k$ to estimate the system disturbance and improve the control performance. Next, an adaptive fuzzy algorithm is designed to compute the variable ${\hat{p}}_k$. Finally, the stability of the adaptive fuzzy sliding mode controller is demonstrated based on the Lyapunov stability condition. Figure 3 illustrates the block diagram of the system controller.

To design the SMC control, the sliding surface is chosen as

10 $$\begin{aligned} s_{k} = e_{k} + \alpha e_{k-1} \end{aligned}$$

In the equation, $\alpha$ denotes a design parameter that satisfies the condition $0< \alpha < 1$, and $e_k$ represents the tracking error between the measured trajectory $y_k$ and its desired value $y_k^*$. Using the single-input single-output model of the PAMs system given in Eq. (9), we can express the tracking error as:

11 $$\begin{aligned} \begin{aligned} e_{k}&= y_{k}^* - y_{k}\\&= y_{k}^*+\sum \limits _{i=1}^{n}{{a}_{i}{y}_{k-i}}-\sum \limits _{j=1}^{m}{{b}_{j}{u}_{k-j}} -p_{k} \end{aligned} \end{aligned}$$

Replace $e_{k}$ from Eq. (11) into Eq. (10), we have

12 $$\begin{aligned} \begin{aligned} s_{k} = y_{k}^*+\sum \limits _{i=1}^{n}{{a}_{i}{y}_{k-i}}&-\sum \limits _{j=1}^{m}{{b}_{j}{u}_{k-j}}-p_{k} +\alpha e_{k-1} \end{aligned} \end{aligned}$$

To guarantee the sliding variable is driven to the sliding surface. We consider the following discrete-time reaching law

13 $$\begin{aligned} \begin{aligned} \Delta s_{k} = s_{k}-s_{k-1} = - K_{sw} s_{k} \end{aligned} \end{aligned}$$

or

14 $$\begin{aligned} s_{k} = (1 + K_{sw})^{-1} s_{k-1} \end{aligned}$$

where $K_{sw} > 0$ is control gain. By replacing $s_{k}$ from Eq. (14) into the Eq. (12), the control signal $u_k$ can be obtained as

15 $$\begin{aligned} \begin{aligned} u_{k}=&\frac{1}{b_1} \left[ \right. y_{k}^* + \sum \limits _{i=1}^{n}{{a}_{i}{y}_{k-i}}-\sum \limits _{j=1}^{m}{{b}_{j}{u}_{k-j}} - p_{k} +\alpha e_{k-1} - (1 + K_{sw})^{-1} s_{k-1} \left. \right] \end{aligned} \end{aligned}$$

This algorithm’s control signal $u_{k}$ includes an uncertain disturbance element $p_{k}$. To implement the control algorithm effectively, it is necessary to accurately determine the value of $p_{k}$. This paper proposes an adaptive fuzzy algorithm for estimating $p_{k}$. This algorithm ensures system stability and enhances the controller’s overall effectiveness. With the estimated value ${\hat{p}}_{k}$ of $p_{k}$, the control signal $u_{k}$ is calculated using the following equation:

16 $$\begin{aligned} \begin{aligned} u_{k}=&\frac{1}{b_1} \left[ \right. y_{k}^* + \sum \limits _{i=1}^{n}{{a}_{i}{y}_{k-i}}-\sum \limits _{j=1}^{m}{{b}_{j}{u}_{k-j}} -{\hat{p}}_{k} +\alpha e_{k-1} - (1+ K_{sw})^{-1} s_{k-1} \left. \right] \end{aligned} \end{aligned}$$

The subsequent subsections will provide a detailed explanation of the proposed adaptive fuzzy algorithm.

### Fuzzy logic system

In this study, a fuzzy system is utilized to estimate the output signal of a system. The fuzzy system operates based on a set of If-Then fuzzy rules related to the known input signals. These rules have the following form:

17 $$\begin{aligned} \begin{aligned} \textit{If}&\\ {}&\left[ s_1(k) \textit{ is } S_1^i,\dots , s_n(k) \textit{ is } S_n^i \right] \\ \textit{then}\\ {}&\left[ {\hat{p}}_k^i = D_k^i \right] \end{aligned} \end{aligned}$$

where $i=1, \dots , N$ with *N* is the number of fuzzy rules of the system; $s_j (k)$ $(j=1, \dots , n)$ are the input signals, ${\hat{p}} _k^i (k)$ are the corresponding output signal.

Due to their high accuracy, the Takagi-Sugeno (TS) fuzzy rules are frequently employed to model nonlinear systems. This study utilizes the Takagi-Sugeno-Kang (TSK) model of order 0. The If-Then fuzzy rules for this model can be represented as follows:

18 $$\begin{aligned} \begin{aligned} \textit{If }\\ {}&\left[ s(k) \textit{ is } S_i \right] \\ \textit{ then }\\&\left[ {\hat{p}}_k^i = D_k^i \right] \text { with }i=1 \dots N \end{aligned} \end{aligned}$$

Assuming that each rule assigns a numerical value to the output $p_k^i = D _k^i$, we can calculate the estimated value of ${\hat{p}} _k$ using a weighted average:

19 $$\begin{aligned} {\hat{p}}_k = \frac{\sum _{i=1}^N w_i \hat{p}_k^i}{\sum _{i=1}^N w_i} \end{aligned}$$

or, similarly,

20 $$\begin{aligned} {\hat{p}}_k = D_k^T W(s_k) \end{aligned}$$

where, $$ D_k$$ = $$[ D_k^1, D_k^2,\dots , D_k^N]^T$$ is the vector containing the attributed values $$D _k^i$$ for rule *i*; $$W(s_k)=[W_1 (s_k),W_2 (s_k),\dots ,W_N (s_k)]^T$$ is a normalized weight vector with $$\displaystyle W_i (s_k)= \frac{w_i}{\sum _{j=1}^N w_j} $$ and $$w_i$$ is the firing strength of each rules. The following subsection will introduce an adaptive law to update the vector $$D_k$$, representing the most accurate approximation of $${p}_k$$. This update will enhance the performance of the system.

### Adaptive law

In order to ensure that the estimated value ${\hat{p}} _k$ accurately reflects the disturbance $p_k$, we introduce an adaptation law to update the parameter vector $D_k$. This adaptation law is given by:

21 $$\begin{aligned} D_{k} = D_{k-1} - \varphi s_{k} W(s_{k}) \end{aligned}$$

where $\varphi$ represents a strictly positive constant associated with the adaptation rate. It is worth noting that:

22 $$\begin{aligned} \Delta D_{k} =D_{k} - D_{k-1} = - \varphi s_{k} W(s_{k}) \end{aligned}$$

Equation (22) also indicates that there is no adaptation occurring when the states are on the sliding surface.

23 $$\begin{aligned} \Delta D_{k} = 0 \textit{ for } s_{k}=0 \end{aligned}$$

### Stability analysis of adaptive fuzzy sliding mode control

In this section, we will demonstrate the stability of the proposed algorithm using the Lyapunov stability condition. This analysis will allow us to determine the range of parameters for the AFSMC controller. Let $D^*_k$ denote the ideal vectors, from which the disturbance value $p_k$ can be calculated as $p_k = D^{*T}_k W(s_k)$. We define the approximation error as follows:

24 $$\begin{aligned} {\tilde{p}}_{k} = p _{k} - {\hat{p}}_{k} \end{aligned}$$

Simultaneously, we consider fuzzy parameter errors

25 $$\begin{aligned} {\tilde{D}}_{k} = D^*_{k} - {D_{k}} \end{aligned}$$

It is obvious that

26 $$\begin{aligned} \tilde{p}_{k} = W(s_{k}) {\tilde{D}}_{k} \end{aligned}$$

Employ the differential calculus with the equation of (Eq. 25) to get the following method

27 $$\begin{aligned} \Delta {\tilde{D}}_{k}= \Delta D^*_{k} -\Delta D_{k} \end{aligned}$$

According to the theory of adaptation rules shown in Eq. (23), when states exist on a sliding surface, no adaptation occurs, as the results $\Delta D^*_{k+1}$ = 0, therefore $\Delta {\tilde{D}}_{k+1}$ is assigned as follows

28 $$\begin{aligned} \Delta {\tilde{D}}_{k}= -\Delta D_{k} = \varphi s_{k} W(s_{k}) \end{aligned}$$

To demonstrate the stability of the system using the proposed algorithm, we will consider the Lyapunov candidate function:

29 $$\begin{aligned} V_k = \frac{1}{2}s_k^2 + \frac{1}{2\varphi } {\tilde{D}}^T _{k} \tilde{D}_{k} \end{aligned}$$

Then,

30 $$\begin{aligned} \begin{aligned} \Delta V_{k}&= V_{k} - V_{k-1} \\&= \frac{1}{2} s_{k}^2-\frac{1}{2} s_{k-1}^2 + \frac{1}{2\varphi } {\tilde{D}}^T _{k} {\tilde{D}}_{k} - \frac{1}{2\varphi } {\tilde{D}}^T _{k-1} {\tilde{D}}_{k-1} \end{aligned} \end{aligned}$$

To compute $\Delta V_k$, we will first examine its first component:

31 $$\begin{aligned} \begin{aligned} \Delta _1&= \frac{1}{2} s_{k}^2-\frac{1}{2} s_{k-1}^2 = \Delta s_{k}s_{k}-\frac{1}{2} (\Delta s_{k})^2 \end{aligned} \end{aligned}$$

In addition,

32 $$\begin{aligned} \begin{aligned} \Delta s_{k} = s_{k} -s_{k-1}= y_{k}^*+\sum \limits _{i=1}^{n}{{a}_{i}{y}_{k-i}}-\sum \limits _{j=0}^{m}{{b}_{j}{u}_{k-j}} -p_{k} +\alpha e_{k-1} -s_{k-1} \end{aligned} \end{aligned}$$

Substituting $u_{k}$ from Eq. (15) into Eq. (32), $\Delta s_{k}$ can be obtained as

33 $$\begin{aligned} \begin{aligned} \Delta s_{k}&= - {\tilde{p}}_{k} + (1+K_{sw})^{-1} s_{k-1} - s_{k-1}\\&= - {\tilde{p}}_{k} - K_{sw}(1+K_{sw})^{-1} s_{k-1}\\&= - {\tilde{p}}_{k} - K_{sw} s_{k} \end{aligned} \end{aligned}$$

Then, Eq. (31) becomes

34 $$\begin{aligned} \begin{aligned} \Delta _1&= (- {\tilde{p}}_{k} - K_{sw} s_{k})s_{k} -\frac{1}{2} (\Delta s_{k})^2\\&= - {\tilde{p}}_{k} s_{k} - K_{sw} s^2_{k} -\frac{1}{2} (\Delta s_{k})^2\\&= -W(s_{k}) \tilde{D}_{k} s_{k} - K_{sw} s^2_{k} -\frac{1}{2} (\Delta s_{k})^2 \end{aligned} \end{aligned}$$

Next, we consider the second part of $\Delta V_{k}$

35 $$\begin{aligned} \begin{aligned} \Delta _2&= \frac{1}{2\varphi } {\tilde{D}}^T _{k} {\tilde{D}}_{k} - \frac{1}{2\varphi } {\tilde{D}}^T _{k-1} {\tilde{D}}_{k-1} \\&= \frac{1}{\varphi }\Delta \tilde{D}_{k} \tilde{D}_{k} - \frac{1}{2 \varphi } (\Delta \tilde{D}_{k})^2\\&=\frac{1}{\varphi }( \varphi s_{k} W(s_{k})) \tilde{D}_{k} - \frac{1}{2 \varphi } (\Delta \tilde{D}_{k})^2\\&= W(s_{k}) \tilde{D}_{k} s_{k} - \frac{1}{2 \varphi } (\Delta \tilde{D}_{k})^2 \end{aligned} \end{aligned}$$

Therefore,

36 $$\begin{aligned} \begin{aligned} \Delta V_{k}&= \Delta _1 + \Delta _2 = - K_{sw} s^2_{k} -\frac{1}{2} (\Delta s_{k})^2 - \frac{1}{2 \varphi } (\Delta \tilde{D}_{k})^2 \le 0 \end{aligned} \end{aligned}$$

Therefore, we have shown that the proposed adaptive fuzzy sliding mode control guarantees the asymptotic stability of the system.

## Experimental results

In this section, we describe a series of experiments that were conducted to assess the performance of the suggested controller with varying trajectories. The main objective of these experiments was to evaluate the controller’s effectiveness under different conditions. We employed the Gaussian membership functions for $S_i$ outlined below to accomplish this.

37 $$\begin{aligned} {\left\{ \begin{array}{ll} u_1 = \displaystyle \frac{1}{1+ e^{5(s+3)}}\\ u_2= \displaystyle e^{-0.25(s+1.5)^2}\\ u_3= \displaystyle e^{-0.25s^2}\\ u_4= \displaystyle e^{-0.25(s-1.5)^2}\\ u_5= \displaystyle \frac{1}{1+e^{-5(s-3)}} \end{array}\right. } \end{aligned}$$

**Figure 4:**
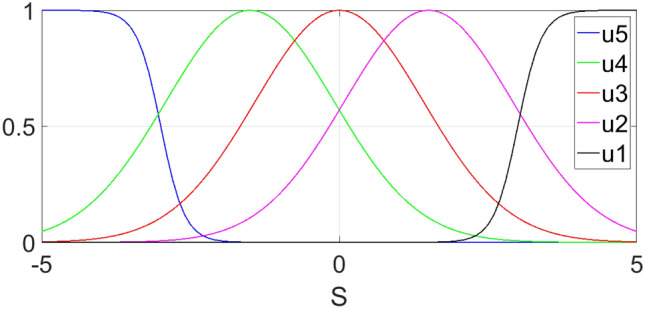
The membership functions of the fuzzy set.

The graph for these membership functions is shown in Fig. 4.

Experiments were conducted with input signals such as sine and various sine waves in two scenarios—with and without load. The control approach was implemented using the LabVIEW/MyRIO toolkit and then embedded into the MyRIO-1900 controller with a sampling time of 5*ms*. The performance of the proposed AFSMC approach and the conventional SMC approach were compared in terms of trajectory tracking. Table 2 presents the parameters for AFSMC and SMC after fine-tuning.

**Table 2:** Parameters of the AFSMC and SMC controllers.

Parameters	$K_{sw}$	$\alpha$	$\varphi$
SMC	0.5	0.1	
AFSMC	0.5	0.1	0.04

### Experimental with sinusoidal trajectories

**Figure 5:**
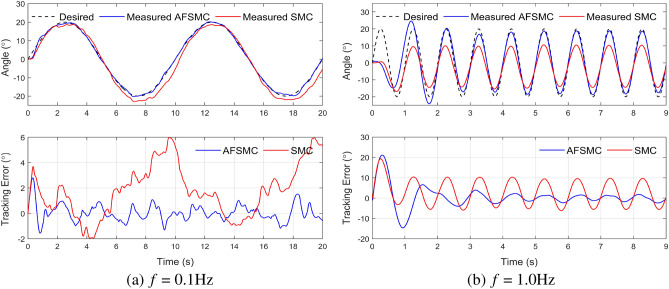
Experimental results for tracking sinusoidal trajectories without load.

The effectiveness of both control strategies, AFSMC and SMC, was first evaluated for the no-load scenario using sine signals with a frequency range of 0.1–1.0 Hz as desired trajectories. The experimental results, shown in Fig. 5, demonstrate that both controllers offer excellent tracking performance, but their effectiveness decreases as the frequency increases. Nevertheless, the AFSMC controller exhibits better tracking performance with a smaller deviation than the SMC controller. Specifically, in the case of 0.1 Hz reference signal, at the steady-state, the SMC controller shows the highest deviation of the dynamic performance at nearly 6.0$^\circ$, whereas the deviation value for AFSMC is much smaller, around 2.2$^\circ$, and consistently converges to 0$^\circ$. In the case of 1.0 Hz reference signal, the maximum error values for SMC and AFSMC are around 10.0$^\circ$ and 4.0$^\circ$, respectively.

**Figure 6:**
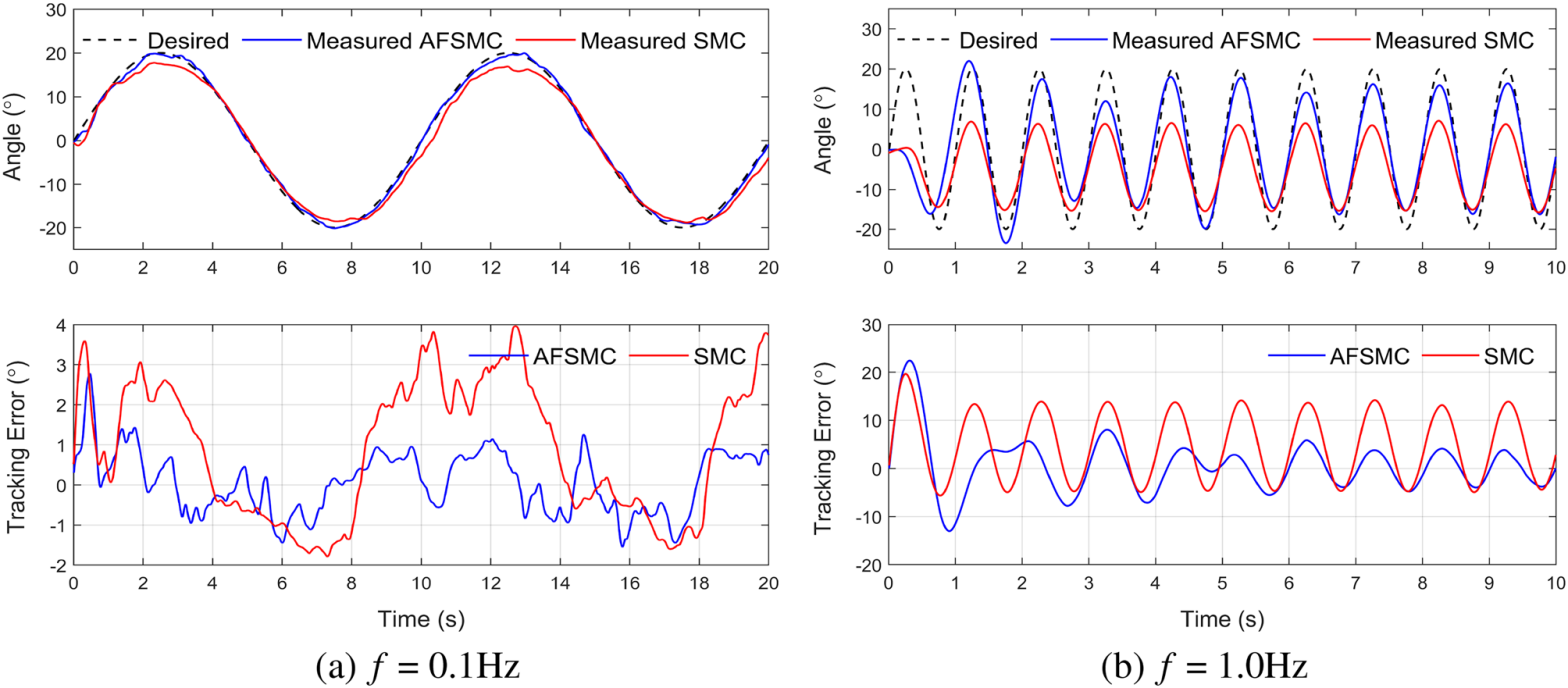
Experimental results for tracking sinusoidal trajectories with an added load of 5 kg.

In the second scenario, a 5 kg load was introduced to the system. This load is equivalent to the leg part of Asian humans^40^. The results of tracking performance and tracking errors are shown in Fig. 6. With 0.1 Hz reference signal, the maximum error values for AFSMC and the SMC at steady-state are around 2.0$^\circ$ and 4.0$^\circ$, respectively. When the frequency of the reference signal increases, the error also increases. With 1.0 Hz reference signal, the maximum error values for AFSMC and the SMC at steady-state are around 4.0$^\circ$ and 10.0$^\circ$, respectively. Remarkably, even with the presence of an external disturbance component, the AFSMC controller continues to demonstrate superior performance compared to SMC as the root mean square tracking error (RMSE) are presented in Table 3. This is due to the accurate estimation of the disturbance element $p_k$, a function of the sliding surface variable $s_k$ determined using an adaptive fuzzy algorithm. Further analysis of the estimation accuracy will be discussed in the next subsection.

**Table 3:** RMSE of two controllers when tracking sinusoidal orbital input.

Frequency	Without load		Load (m = 5 kg)	
	AFSMC	SMC	AFSMC	SMC
0.1 Hz	0.71	1.15	0.74	1.18
0.5 Hz	2.23	3.92	2.68	4.21
1.0 Hz	3.13	5.40	3.48	6.64

### Experimental with mixed sinusoidal trajectories.

Besides using sinusoidal trajectories, the tracking performance of AFSMC and SMC controllers are also evaluated using a mixed sinusoidal reference trajectory as described by the following equation: $\theta (t)=20\sin {2\pi f} + 12.8\sin {\pi f}$. The reference signal’s basis frequency *f* ranges from 0.1 to 0.8 Hz in this experiment.

**Figure 7:**
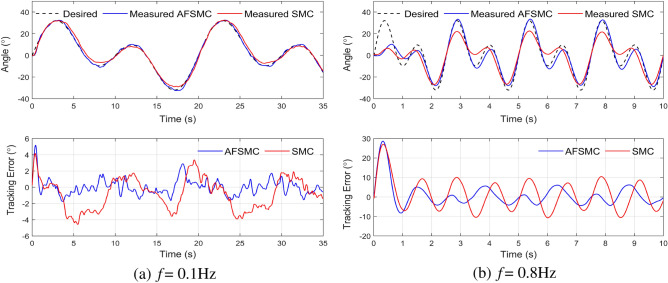
Experimental results for tracking a mixed-sine trajectory without a load.

The first scenario involves the unloaded system, and the tracking performance of the two controllers is shown in Fig. 7. With 0.1 Hz reference trajectory, the maximum steady-state error for SMC is approximately 4.5$^\circ$, whereas AFSMC is much lower at around 2.0$^\circ$. With 0.5 Hz reference trajectory, the maximum steady-state error for SMC and AFSMC is approximately 9.8$^\circ$ and 4.1$^\circ$, respectively. Additionally, AFSMC’s tracking performance remains effective with 0.8 Hz reference trajectory. Notably, AFSMC exhibits orbital tracking with a maximum error of approximately 6.5$^\circ$, while SMC’s value is around 10.5$^\circ$. This confirms that SMC is less capable of adapting to high-frequency orbitals, particularly with complex trajectories. On the other hand, AFSMC continues to perform well when tracking complicated trajectories such as mixed sinusoidal signals. This experiment further demonstrates the effectiveness of the adaptive fuzzy algorithm in compensating for the systematic noise of the nonlinear model, i.e., the artificial muscle system.

**Figure 8:**
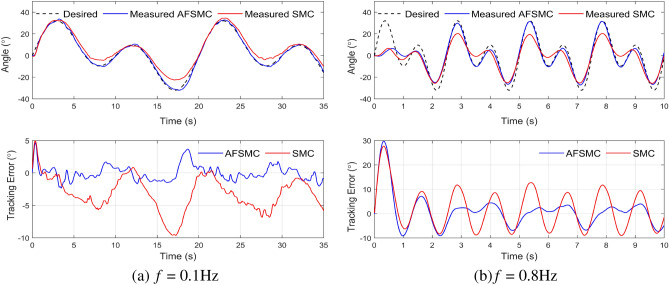
Experimental results for tracking a mixed-sine trajectory with a load (m = 5 kg).

In the loaded system scenario, both the SMC and AFSMC controllers experience a performance decrease. However, the AFSMC controller demonstrates superior performance due to its ability to adapt to the system’s disturbances. With 0.8 Hz reference trajectory, the maximum steady-state error values for SMC and AFSMC were approximately 15.0$^\circ$ and 8.0$^\circ$, respectively. The control quality of both SMC and AFSMC controllers is illustrated in Fig. 8, while RMSE for both controllers in both loaded and unloaded test scenarios are summarized in Table 4.

**Table 4:** RMSE of two controllers when tracking a mixed-sine trajectory.

Basis frequency *f*	Without load		Load (m = 5 kg)	
	AFSMC	SMC	AFSMC	SMC
0.1 Hz	0.99	2.07	1.18	4.13
0.5 Hz	4.26	6.43	4.17	7.34
0.8 Hz	6.14	7.99	6.76	8.38

One of the main benefits of the proposed control approach is its ability to adapt to external disturbances effectively. To demonstrate this, the system was first operated to track a mixed sinusoidal signal with a basic frequency of $f=0.5$ Hz without any load until reaching steady state. Next, a load was suddenly added to the system, and data was collected for a total of 45 s for further analysis. The results showed that the PAMs controlled by AFSMC had superior adaptation to the moment of load shift compared to SMC.

**Figure 9:**
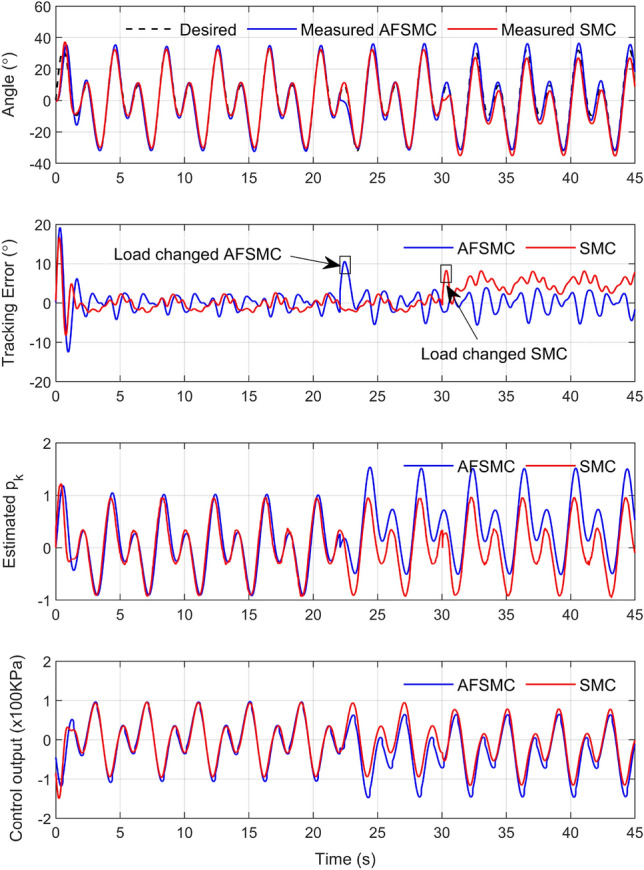
The investigation of the disturbance estimation when the load is suddenly added.

Figure 9 illustrates the difference between the AFSMC and SMC controllers when a load is suddenly added to the system. The time from the system startup when the load is added is around 23 s and 30 s for the AFSMC and SMC controllers, respectively. Both controllers exhibit slight fluctuations in their trajectories. However, the AFSMC quickly returns to track the desired value by manipulating its control output. This is due to the estimation of the disturbance component $p_k$ using the adaptive fuzzy algorithm with immediate adaptation. In contrast, the SMC cannot accurately estimate $p_k$ as the AFSMC does. As a result, the control output of the SMC slightly changes and cannot return to track the desired trajectory.

## Conclusion

This work proposes an adaptive fuzzy sliding mode control approach for the PAM-based system to improve tracking performance by estimating and compensating for external disturbances. The disturbance component $p_k$ is estimated using the Takagi-Sugeno fuzzy algorithm, and the output variable ${\hat{D}}$ values are updated automatically by an adaptive law. The proposed AFSMC controller is evaluated through experiments with sine signal inputs ranging from 0.1 to 1.0 Hz. The results show improved tracking accuracy compared to the traditional sliding mode control approach. For instance, the RMSE value with load at 0.5 Hz is 2.68$^\circ$ for AFSMC and 4.21$^\circ$ for SMC. Moreover, when a load is suddenly added to the system, the AFSMC controller demonstrates better adaptability than the SMC approach. The AFSMC controller quickly returns to track the desired value by manipulating its control output, while the SMC cannot reach a highly accurate estimation of $p_k$ and its control output slightly changes, resulting in an inability to return to the desired trajectory. The experimental results demonstrate that the proposed AFSMC approach adapts to external disturbances more than the traditional SMC approach. However, the proposed AFSMC approach shows weakness in the transitional period, where chattering may occur as ${\hat{p}} _k$ approaches ${\hat{p}} _k^*$. Further studies are needed to address this issue and improve the quality of the AFSMC controller.

## Data Availability

The datasets used and/or analyzed during the current study are available from the corresponding author upon reasonable request.
